# Functional Responses of Three Neotropical Mirid Predators to Eggs of *Tuta absoluta* on Tomato

**DOI:** 10.3390/insects7030034

**Published:** 2016-07-12

**Authors:** Joop C. van Lenteren, Lia Hemerik, Juracy C. Lins, Vanda H. P. Bueno

**Affiliations:** 1 Laboratory of Entomology, Wageningen University, P.O. Box 16, Wageningen 6700 EH, The Netherlands; 2 Biometris, Department of Mathematical and Statistical Methods, Wageningen University, P.O. Box 16, Wageningen 6700 AA, The Netherlands; lia.hemerik@wur.nl; 3 Laboratory of Entomology, University Center of Várzea Grande, Várzea Grande MT 78118-900, Brazil; juracylins@yahoo.com.br; 4 Laboratory of Biological Control, Department of Entomology, Federal University of Lavras, P. O. Box 3037, Lavras MG 37200-000, Brazil; vhpbueno@den.ufla.br; 5 Laboratory Chemical Ecology and Insect Behavior, Department of Entomology and Acarology, Escola Superior de Agricultura Luiz de Queiroz (ESALQ/USP), Piracicaba SP 13418-900, Brazil

**Keywords:** Miridae, *Campyloneuropsis infumatus*, *Engytatus varians*, *Macrolophus basicornis*, biological control, zoophytophagy, tomato borer

## Abstract

*Tuta absoluta* (Meyrick) has quickly developed into a significant tomato pest worldwide. While the recently found mirid predators *Macrolophus basicornis* (Stal), *Engytatus varians* (Distant) and *Campyloneuropsis infumatus* (Carvalho) of this pest are able to establish and reproduce on tomato, biological knowledge of these mirids is still limited. Here we describe the functional response of the three mirid predators of the tomato pest *T. absoluta* when offered a range of prey densities (four, eight, 16, 32, 64, 128 and 256 eggs) during a 24 h period inside cylindrical plastic cages in the laboratory. *Engytatus varians* and *M. basicornis* showed a type III functional response, whereas *C. infumatus* showed a type II functional response. At the highest prey densities, *C. infumatus* consumed an average of 51.0 eggs, *E. varians* 91.1 eggs, and *M. basicornis* 100.8 eggs. Taking all information into account that we have collected of these three Neotropical mirid species, we predict that *M. basicornis* might be the best candidate for control of the tomato borer in Brazil: it has the highest fecundity, the largest maximum predation capacity, and it reacts in a density-dependent way to the widest prey range.

## 1. Introduction

Due to the ecosystem services provided by parasitoids, predators, and pathogens, populations of potentially damaging herbivorous insects are often maintained at tolerable levels [1,2]. Where agroecosystems prevent natural enemies from sufficiently reducing pest numbers, for example due to lack of necessary resources such as food or hiding places or due to killing beneficial organisms with pesticide sprays, natural enemies need to be reintroduced in biological control programs. In order to decide what kind of biological control can be used—inundative, seasonal inoculative or classical/inoculative biological control [3]—it is important to know whether and how a natural enemy is able to bring pest numbers down to non-damaging densities. For classical and seasonal inoculative releases a natural enemy needs to possess strong enough numerical and functional responses, whereas for inundative releases such responses are appreciated but not necessary. The numerical response is the change in predator density as a function of the change in prey density [4]: as a result of an increase in prey density, predators have more food and offspring and, thus, also increase in numbers, and vice versa. The functional response is the change in an individual predator’s rate of prey consumption with the change in prey density. Together, the numerical response (a reproduction response) and the functional response (a predation response) determine the pest population regulation capacity of a predator [5].

Holling [6] described three types of functional responses (see Section 2.3). Type I functional responses result in density-independent prey killing, type II in a negatively density-dependent response where with increasing prey density a decreasing percentage of prey is consumed, and type III in a positively density-dependent response, where, over a certain range of increasing prey densities, an increasing percentage of prey is killed. In the literature, type II functional responses are most often reported [6,7] though there are cases of type III reported, and, interestingly, sometimes one species of predator shows different types of functional responses when encountering different types of prey. For example, the mirid predator *M. pygmaeus* shows a type II response when exposed to various densities of the aphid *Myzus persicae* (Sulzer) (Hemiptera: Aphididae) [8], but a type III response when offered different densities of *Trialeurodes vaporariorum* (Westwood) (Hemiptera: Aleyrodidae) [9].

We are currently evaluating native Brazilian predators of the tomato borer, *Tuta absoluta* (Meyrick) (Lep.: Gelechiidae), a major pest of tomato which is spreading all over the world since its accidental introduction into Spain in 2006 [10]. Recent reviews indicate that many natural enemy species of *T. absoluta* are available [11,12], but very few of these have been tested under field conditions or are commercially used [3]. An exception is formed by two mirid predators, *Nesidiocoris tenuis* (Reuter) and *Macrolophus pygmaeus* (Rambur) (Hem.: Miridae), that are providing effective control of *T. absoluta* in Mediterranean Europe [13,14]. Due to recent developments under the Convention of Biological Diversity, in particular as an effect of the acceptance of the Nagoya Protocol [15] on Access to Genetic Resources and the Fair and Equitable Sharing of Benefits, these European mirids cannot easily be imported and released outside their area of origin. We have, therefore, decided to collect and evaluate natural enemies of *T. absoluta* in an important tomato production area in Brazil (Minas Gerais) and recently reported the first promising results about the mirid predators *Macrolophus basicornis* (Stal), *Engytatus varians* (Distant) and *Campyloneuropsis infumatus* (Carvalho) (Hem.: Miridae) when exposed to the tomato borer on tomato. These predators appeared able to walk and reproduce on tomato plants [16], prey on eggs and larvae of *T. absoluta*, and they exhibited high fecundity and survival rates on tomato [17].

Life table data of these three mirid species showed that the intrinsic rate of population increase (r_m_) is in the order of 0.10–0.11 [18], while the r_m_ of the pest *T. absoluta* shows values between 0.16 and 0.12 on susceptible and partially resistant tomato lines, respectively [19]. As the r_m_ values are in the same order of magnitude, the intrinsic rate of population increase of predators and pests is similar. The effectiveness of the mirid predators is, therefore, expected to mainly depend on their functional responses.

In this paper, we describe the results of a study concerning the functional responses of three mirid predators, *M. basicornis*, *E. varians* and *C. infumatus*, when offered a range of prey densities (four, eight, 16, 32, 64, 128 and 256) of the tomato pest *T. absoluta* during a 24 h period inside cages in the laboratory. We intend to use the results of the functional response study to preselect candidates for biological control of the tomato borer.

## 2. Material and Methods

### 2.1. Insect Rearing

*Tuta absoluta* individuals collected in experimental tomato fields on the campus of the Federal University of Lavras (Minas Gerais, Brazil) were taken into the laboratory and reared in a climate room at 25 ± 2 °C, 60% ± 10% R.H. and a 12 h photo phase inside cages (90 cm × 70 cm × 70 cm) on tomato plant cv. Santa Clara. New tomato plants were introduced regularly to the cage with the stock colony of *T. absoluta.* The predators *C. infumatus, E. varians* and *M. basicornis* were field collected in Ribeirão Vermelho, State of Minas Gerais (Brazil) on tobacco (*Nicotiana tabacum* L. cv TNN) [16] and reared according to Bueno et al. [17] at the same climate conditions as the pest. The predators were kept inside acrylic cages (60 cm × 30 cm × 30 cm) with a tobacco plant as oviposition substrate and eggs of *Ephestia kuehniella* (Zeller) (Lepidoptera: Pyralidae) as food; for details see [18].

### 2.2. Determination of Functional Response

Tomato seedlings (cv Santa Clara, approximately 10 cm in height and with three to four leaves, grown in a greenhouse under natural conditions on organic substrate (75% of *Pinus* rusk and 25% of vermiculate) and without pesticide applications) were infested with different densities of *T. absoluta* eggs and offered to the predators. The seedlings were grown in plastic cups (50 mL) and after *T. absoluta* infestation, they were put inside cylindric plastic cages (20 cm high, 10 cm diameter) covered with organza tissue on the top. Females of each predator species from 2 to 7-days-old were used as preliminary testing showed that predation rates are stable during this period [20]. Predators were starved for 24 h before the tests. Prey densities to be offered were determined through preliminary tests to ensure that maximum levels of predation could be obtained by each predator, which resulted in densities of four, eight, 16, 32, 64, 128 and 256 eggs of *T. absoluta*/tomato seedling for *E. varians* and *M. basicornis*. As *C. infumatus* consumed fewer eggs in the preliminary tests than the other two mirids, the density of 256 eggs was not evaluated for this species. A single female of each predator was released in each cylindric cage and the number of consumed eggs was counted after 24 h. The experiment was carried out in a climate room at 25 ± 3 °C, 60% ± 10% R.H. and a 12 h photo phase. Prey was not replaced during the tests, as we offered an excess of prey. The tests consisted of 25 replicates for each prey density and for each mirid species. The number of eggs eaten was determined by counting dead and shriveled eggs under a stereomicroscope at a magnification of 40×; completely consumed eggs are transparent and show a ruptured chorion caused by the stylets of the mirids. A control treatment without predators was carried out, consisting of 10 arenas for each prey density to determine the natural mortality by counting the dead, shriveled eggs which, in this case may still be yellow or dark colored.

### 2.3. Data Analysis

Experimental data were fitted to functional response curves as defined by Holling [6], namely linear (Holling type I), monotonic increasing unto a plateau (Holling type II) and sigmoidal increasing to a plateau (Holling type III). We assumed that these functional responses were constructed of a probability *p*(*x*) for an individual of being eaten at density *x* times the density *x* (Equation (1)). This *p*(*x*) is the success probability of a binomial distribution and is density dependent in case of Holling type II and III [21].

(1) $$f(x)=p_{i}(x)x$$

The probability is different for each of the Holling types (see Equation (2a)–(2c)). The parameters $a_{i}$ (*i* = I, II, III) represent a scaling and $h_{i}$ (*i* = II, III) is the density at which the probability to be eaten is 0.5. We fitted the models to the data sets by likelihood maximization.

(2a) $$p_{I}(x)=a_{I}$$

(2b) $$p_{II}(x)=\frac{a_{II}}{h_{II}+x}$$

(2c) $$p_{III}(x)=\frac{a_{III}x}{h_{III}^{2}+x^{2}}$$

Model selection was performed with Akaike’s information criterion [22] corrected for small sample sizes (AICc, Equation (3)). The best model is the one with minimum AICc. The relative support (quantified using the AICc weight, see Equation (4a) and (4b)) is given in the results.

(3) $$AICc=-2\ln(L)-2k+\frac{2k(k+1)}{n-k-1}$$

where *L* is the maximum likelihood, *k* is the number of parameters in the model and *n* is the sample size.

(4a) $$\Delta AICc_{i}=AICc_{i}-\min_{i\in \{I,II,III\}}(AICc_{i})$$

(4b) $$AICc\;\text{weight}=\frac{\text{exp}(-\Delta AICc_{i}/2)}{\sum_{i}\text{exp}(-\Delta AICc_{i}/2)}$$

## 3. Results

Prey mortality in absence of the predators ranged from 0% to 1.12% (*n* = 12,680 eggs), thus reflecting little natural or manipulation mortality and, consequently, data did not have to be corrected. Two of the three mirid predators, *E. varians* and *M. basicornis*, show a type III functional response: the percentage prey consumed at densities four and eight is relatively low, then increases, and finally levels off and decreases at a density over 64 *T. absoluta* eggs. *Campyloneuropsis infumatus* shows a type II functional response and the percentage prey eaten by this predator decreases with the increasing prey density. Not all individuals of the three species found prey at the lowest prey density (four eggs/leaf) and 20% of *E. varians* even did not find a single prey at a density of eight eggs/leaf. At the highest prey densities, *C. infumatus* consumed an average of 51.0 ± 4.50 eggs (minimum six, maximum 87 *T. absoluta* eggs), *E. varians* an average of 91.1 ± 6.07 eggs (minimum 32, maximum 147 *T. absoluta* eggs) and *M. basicornis* an average of 100.8 ± 3.25 eggs (minimum 63, maximum 119 *T. absoluta* eggs).

**Figure 1:**
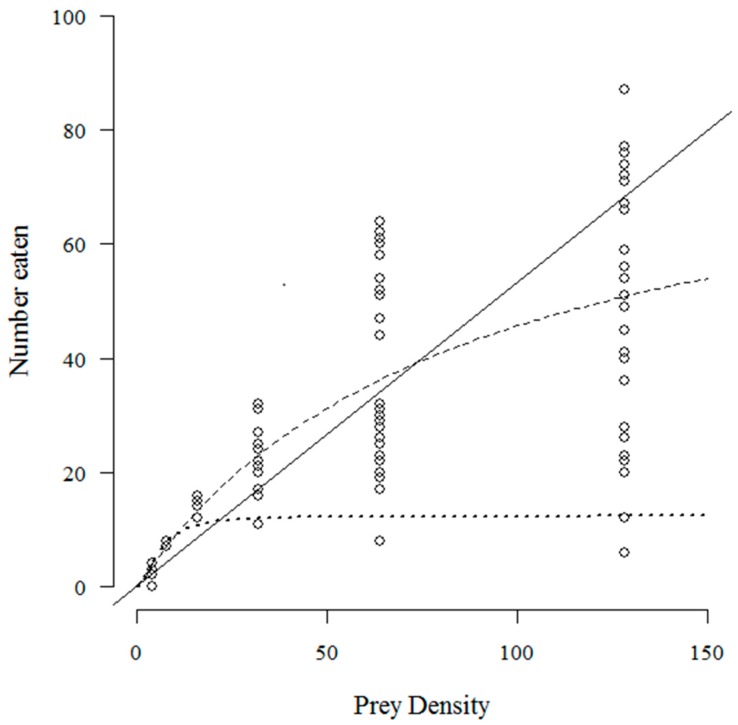
Number of *T. absoluta* eggs eaten during 24 h by the mirid predator *C. infumatus* when offered four, eight, 16, 32, 64 and 128 eggs. The dashed curve—a type II functional response—describes the data best. The drawn line represents the fit for a type I response, and the dotted curve for a type III response. The open circles represent the values found for each replicate; 25 replicates were performed for each prey density.

**Figure 2:**
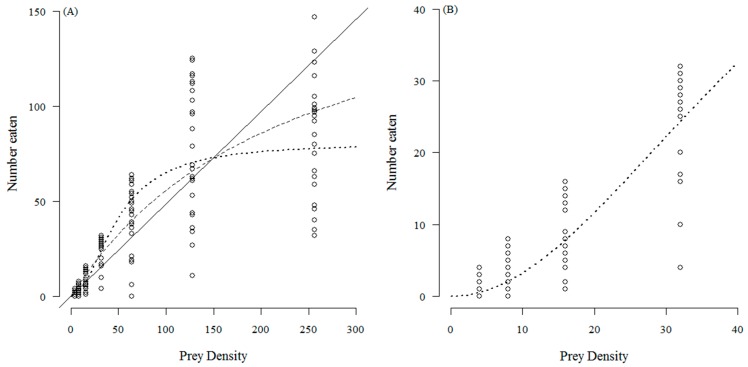
Number of *T. absoluta* eggs eaten during 24 h by the mirid predator *E. varians* when offered four, eight, 16, 32, 64, 128 and 256 eggs. The dotted curve—a type III functional response—describes the data best. The drawn line represents the fit for a type I response, and the dashed curve for a type II response. The open circles represent the values found for each replicate; 25 replicates were done for each prey density; (**A**) shows the full range of tested densities; (**B**) shows the curve at the four lowest densities to confirm the sigmoidal functional response.

**Figure 3:**
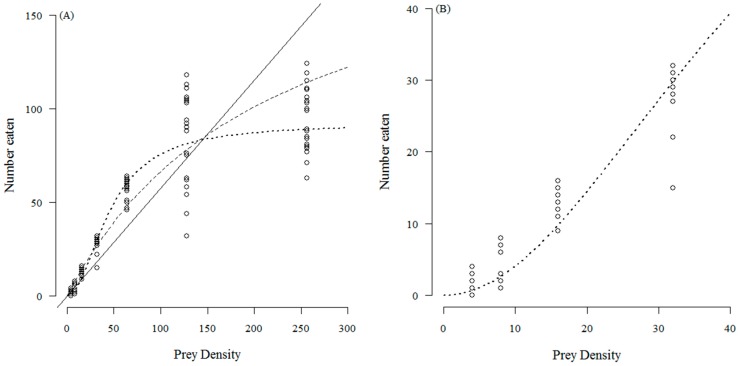
Number of *T. absoluta* eggs eaten during 24 h by the mirid predator *M. basicornis* when offered four, eight, 16, 32, 64, 128 and 256 eggs. The dotted curve—a type III functional response—describes the data best. The drawn line represents the fit for a type I response, and the dashed curve for a type II response. The open circles represent the values found for each replicate; 25 replicates were done for each prey density; (**A**) shows the full range of tested densities; (**B**) shows the curve at the four lowest densities to confirm the sigmoidal functional response.

The number of *T. absoluta* eggs eaten with increasing prey density by *C. infumatus* is shown in Figure 1 and was best described by the Holling type II response (see Table 1a). Alternately, the Holling type III response curve fitted best to the functional response of *E. varians* (see Table 1b). The sigmoid shape of the response in Figure 2a is not easy to see at the low densities, but it becomes visible when we zoom in to the density range two to 32 (Figure 2b). Similar to *E. varians*, *M. basicornis* also exhibited a type III functional response (Table 1c), with the sigmoid shape of the curve being apparent at prey densities ranging from two to 32 *T. absoluta* eggs (Figure 3b).

## 4. Discussion

Differences in the shape of functional responses of predators may depend on many variables such as prey species, size and appearance [23,24], prey distribution [25], availability of alternative prey, the predator’s age and hunger level [6], the plant structure on which the prey occurs [26], the temperature [27], the experimental conditions [25,28], and the type of analysis applied to estimate the response [29]. Addressing the last point, we used a straightforward way to fit the functional response curves and then applied model selection based on Akaike’s information criterion, which is a powerful method to select the best-fitting curve [25]. We kept the following variables constant: prey species, size and appearance, availability of alternative prey (not present in our experiments), predator’s age and hunger level, plant structure, temperature and experimental conditions. Prey density varied, of course, and this influenced prey distribution on the leaflets, which was random at low densities and became more or less regular at high densities. In some predatory Heteroptera (1) plant feeding is obligatory for development and reproduction, in other species (2) it is facultative and may result in faster development and higher reproduction, and in other species still (3) it is facultative and does not contribute to faster development and/or higher reproduction, but can result in survival in the absence of prey [30,31]. The three mirid species studied here all show facultative phytophagous behavior (type 3); they are unable to complete development purely on plant food and they show higher mortality with only plant food than when prey is also available [32].

The type II functional response which we found for *Campyloneuropsis infumatus* has been reported for two other mirids, *Dicyphus tamaninii* and *M. pygmaeus*, when offered, among others, *Frankliniella occidentalis* (Pergande) [33], *Aphis gossypii* [34], *Myzus persicae* [8,35], and *Tetranychus urticae* [8]. A type II functional response does not have stabilizing properties, since the percentage of attacked prey decreases with increasing prey density. At the lowest prey densities we tested (4, 8, 16 and 32), the numbers of prey killed were similar to that of *E. varians* and *M. basicornis*, but at higher prey densities *C. infumatus* kills much less prey. Predators showing a type II response can be used in inundative biological control programs that intend to obtain a direct pest reduction effect, but do not aim at permanent pest control over many years such as in classical biological control [3]. The desired control effect can be obtained by releasing an amount of predators that is large enough to kill the estimated number of prey present in the crop.

Type III functional responses were initially found only for vertebrate predators and were explained by the latter’s capability to develop a searching image when encounters with a certain type of prey increased, and in this way the number of prey attacked rose faster than the prey density [5]. Type III responses were mainly thought to be based on learning behavior of predators. However, later, functional responses for invertebrate predators and parasitoids were also found [28,36]. The lack of earlier reports of type III responses for invertebrates could partly be explained by experimentation errors. Use of small experimental containers or long exposure times resulted in very high probabilities of finding prey at even the lowest densities. Also, the predators are more or less forced to stay on the substrate on which the prey is offered. When larger containers or shorter observation periods were used, type III responses were indeed found [28,36]. Enkegaard et al. [9], who found a type III response for the mirid predator *M. pygmaeus*, used relatively large containers compared to the Petri dishes and small vials in experiments with mirids where type II responses were found [8,33,34,35]. Enkegaard et al. [17] saw the predators regularly leaving the tomato leaves at low prey densities, an observation earlier described for a parasitoid showing a type III functional response [36]. We also used relatively large containers and seedlings with several leaves, providing opportunities for predators to leave the plant substrate, which we did regularly observe at low prey densities. Today, there are several explanations available for the occurrence of type III responses in invertebrates. They can, for example, result from very simple changes in behavior, such as a change from a fixed initial searching time (or giving-up time) when no prey is found to an increase in searching time after a prey has been found [36,37,38].

Interpretation of functional response results is often difficult and their meaning for estimating the biological control capacity of natural enemies is limited. However, predation rates obtained for a range of prey densities provide at least some insight into a predator’s ability to reduce pest densities. For the specific case of *T. absoluta*, we know tomato borer females lay 10–17 eggs per day on Brazilian tomato lines during their 15 day oviposition period at 25 °C [19], and up to 146 borer eggs per plant have been found on a tomato crop in Brazil [39]. Based on the predation rates of the three mirids and the fecundity data for *T. absoluta*, we may speculate that they are able to bring pest numbers down considerably, and maybe sufficiently. However, many more pests may occur on tomato. In Brazil, six other lepidopteran pests, whitefly, dipteran leafminers, thrips, aphids and mites are often found [17], and our first prey acceptance and preference studies with the three mirids showed that they easily accept the other lepidopterans, whiteflies, mites, and some (but not all) aphid species; we have not yet studied the acceptance of dipteran leafminers and thrips [40]. The presence of these pests will influence the predation of *T. absoluta*, but as the tomato borer appeared to be one of the preferred prey species in our tests, we do not expect the mirids will ignore the tomato borer when other pests are present. Without further knowledge about temporal occurrence, distribution and densities of these other pests, it is difficult to speculate about quantitative effects of combinations of pests on the predation of *T. absoluta* eggs by mirids.

For *E. varians* and *M. basicornis* we do not know yet on which behavioral mechanism the type III functional response is based. We do know, however, that this response results in an increasing percentage of prey killed at a certain range of prey densities, and that over this range the response may act as a stabilizing factor. The number and, more importantly, the percentage prey killed increases up to a density of 32 for *E. varians* and even up to 64 for *M. basicornis*. Thus, these two predators are potentially capable of bringing down pest populations in a prey density range of up to 32 or 64 *T. absoluta* eggs per three to four tomato leaves, densities which are similar to or higher than those usually found (1–10/leaf) in a tomato crop [39]. Taking all the information we have collected about these three Neotropical mirid species into account [16,17,18,19,20,40], we hypothesize that *M. basicornis* might be the best candidate for the control of the tomato borer in Brazil: it has the highest fecundity [18], the largest maximum predation capacity (this study), and it reacts in a density-dependent way to the widest prey range (this study). The next step in our project will be to test this prediction in a greenhouse experiment.

## 5. Conclusions

Three recently collected native Brazilian mirid species consume large amounts of *T. absoluta* eggs during 24 h exposure to prey on tomato plants. One of the species, *C. infumatus*, shows a type II functional response and also killed the lowest number of prey eggs, i.e., 51 eggs/day, suggesting that this might be the least suitable of the these mirids for control of *T. absoluta*. The other two species, *E. varians* and *M. basicornis,* show a type III functional response and kill on average 91 and 101 prey eggs/day. As *M. basicornis* kills most prey per 24 h and shows a density-dependent response over the widest prey range, this species might be the best candidate for control of *T. absoluta*.

## Figures and Tables

**Table 1:** Results of the model selection with Akaike’s information criterion and parameters for the best model for each predator species.

Mirid Species	AICc	ΔAICc	AICc Weight	*a*	*h*
(a) *C. infumatus*					
Holling type I	2715.1	903.2	≈0		
Holling type II	1811.9	0	≈1	84.87	85.83
Holling type III	5292.2	3480.3	≈0		
(b) *E. varians*					
Holling type I	4675.6	1083.2	≈0		
Holling type II	3631.6	39.2	≈0		
Holling type III	3592.4	0	≈1	80.53	48.54
(c) *M. basicornis*					
Holling type I	4351.0	2288.2	≈0		
Holling type II	2163.6	100.8	≈0		
Holling type III	2062.8	0	≈1	91.81	46.16
